# The Role of APTES as a Primer for Polystyrene Coated AA2024-T3

**DOI:** 10.3390/mi15010093

**Published:** 2023-12-31

**Authors:** John Halford, Cheng-fu Chen

**Affiliations:** Department of Mechanical Engineering, University of Alaska Fairbanks, Fairbanks, AK 99775-5905, USA; jhhalfordiv@alaska.edu

**Keywords:** vapor deposition, coating, primer, polystyrene, APTES, silanes, AA2024-T3, corrosion, electrochemical impedance spectroscopy, surface energy

## Abstract

(3-Aminopropyl)triethoxysilane (APTES) silane possesses one terminal amine group and three ethoxy groups extending from each silicon atom, acting as a crucial interface between organic and inorganic materials. In this study, after APTES was deposited on the aluminum alloy AA2024-T3 as a primer for an optional top coating with polystyrene (PS), its role with regard to stability as a protection layer and interaction with the topcoat were studied via combinatorial experimentation. The aluminum alloy samples primed with APTES under various durations of concentrated vapor deposition (20, 40, or 60 min) with an optional post heat treatment and/or PS topcoat were comparatively characterized via electrochemical impedance spectroscopy (EIS) and surface energy. The samples top-coated with PS on an APTES layer primed for 40 min with a post heat treatment revealed excellent performance regarding corrosion impedance. A primed APTES surface with higher surface energy accounted for this higher corrosion impedance. Based on the SEM images and the surface energy calculated from the measured contact angles on the APTES-primed surfaces, four mechanisms are suggested to explain that the good protection performance of the APTES/PS coating system can be attributed to the enhanced wettability of PS on the cured APTES primer with higher surface energy. The results also suggest that, in the early stages of exposure to the corrosion solution, a thinner APTES primer (deposited for 20 min) enhances protection against corrosion, which can be attributed to the hydrolytic stability and hydrolyzation/condensation of the soaked APTES and the dissolution of the naturally formed aluminum oxide pre-existing in the bare samples. An APTES primer subjected to additional heat treatment will increase the impedance of the coating system significantly. APTES, and silanes, in general, used as adherent agents or surface modifiers, have a wide range of potential applications in micro devices, as projected in the Discussion section.

## 1. Introduction

Silanes and silane esters have demonstrated considerable promise as corrosion-resistant adhesion promoters [1] and undercoats [2] or for use in paint emulsions [3]. APTES, a reactive silane ester (trialkoxysilane) known for its grafting capabilities, enhances compatibility between minerals and organic polymers. APTES can be applied for surface functionalization as a primer or for the surface modification of nanoparticles to form nanocomposites; both applications involve using APTES as a surface-priming agent for top coating with functionalized polymers. Including additional compounds, including magnetite nanoparticles for their magnetic properties, silicate nanoparticles for enhanced strength, titanium dioxide nanoparticles for UV resistance, polyaniline for conductivity, and graphene/carbon nanotubes for reinforcement, further tailors nanocomposites’ functionalities [4]. Determining how APTES can be grafted for surface modification or priming is crucial to making a stable and durable coating system.

APTES has been examined for use as a primer compound or as a compatibilizer between organic and inorganic materials [5]. As a primer or adhesion promoter, APTES has a terminal amine group that is less sterically constrained to the silanols, as can be demonstrated by the chair conformational isomer nitrogen–oxygen distance of 0.245 nm in hydrolyzed APTES [5]. The spatial relationship of these functional groups facilitates pH-dependent hydrolysis and the formation of denser networks of oligomers [6]. Trialkoxysilanes hydrolyze quickly (in minutes), forming free silanols, but tend to condense much more slowly (in the order of hours). The networking stability of APTES is governed by the competition between the hydrolysis and condensation mechanisms, which can be optimized at a pH value of around 6, given that the rates of condensation and hydrolyzation have minima at pH 4 and 7.5, respectively, for a typical silane [5,7]. Accessibility to water is a key factor in determining the performance of APTES as a primer for the bonding of organic/inorganic materials. Although silane coatings often enhance adhesion, silanes such as γ-aminopropyl silane (γ-APS) perform poorly when immersed in water and may fail wet adhesion tests [2]. Silanes like APTES do not bond to the Cu-rich sites on the surface of aluminum alloys, which are highly susceptible to local pitting [1]. Therefore, silane coatings are typically used as a primer for additional top coatings. In this context, silane compounds are recognized for their potential as effective adhesion promoters between organic coatings and oxidized metal surfaces.

Applications of silanes can be classified based on their grafting functionality on inorganic surfaces [8,9] or their integrity with regard to top-coating polymers for resistance to coating rupture and disbonding [10,11,12]. A TiO_2_-APTES nanocomposite was scrutinized for its interfacial stability and failure [8]. PS has emerged as a potential topcoat due to its efficient water repellency and relatively low surface energy of 31 mJ/m^2^ [13]. The non-polar and chemically inert characteristics of PS, owing to its aromatic benzene ring, make it resistant to reactions with acids and bases, simplifying corrosion analysis [13]. PS in a solution form can be directly spin-coated onto the surface of metals like aluminum alloys. However, its effectiveness as a protective coating is limited by its poor adhesion to metals and susceptibility to thin-film rupture when present as a dried film. The dewetting of PS on monolayered APTES was addressed in [12]. Zhang recently addressed the thin-film porosity and integrity issues relating to PS [14] by developing a densely compacted polystyrene/TiO_2_ nanocomposite coating. A PS layer can be applied as a topcoat over a silane [15]. This approach effectively mitigates the individual drawbacks of silane and PS coatings. The top-coated PS acts as a barrier, safeguarding the underlying silane primer from water attacks. Simultaneously, the silane primer plays a dual role as an additional protective layer for the metal substrate and an adhesive agent, indirectly binding the PS to the substrate.

However, the role the stacked silane/polymer coating plays after coating failure has not been well characterized, except in a few works [12,15,16]. Chen [15] applied an APTES/toluene solution to a hydroxylated AA2024-T3 surface. The sample surface had been grafted with the hydroxyl group before being soaked in a diluted APTES/anhydrous toluene solution to enable the formation of siloxane bonds or hydrogen bonds with APTES; however, through soaking, the primed APTES layer became thicker and less uniform than a monolayer [6,17], adding undesired complexity in the networked structure. An APTES primer prepared via solvent-based soaking also exhibits a physisorption behavior, yielding a less-stable networked structure because the un-bonded/loosened ethoxy groups hidden in the cured APTES can migrate to the network surface and then react with water for hydrolyzation and subsequent condensation, both of which will change the thickness of the cured APTES layer [17].

In this work, the aluminum alloy AA2024-T3 was chosen for priming with APTES and the subsequent topcoat with PS to characterize the role of the APTES primer in the stacked coating system. AA2024-T3 has been extensively addressed in the literature regarding surface treatments [18]. In this study, the method of concentrated vapor deposition in low-vacuum conditions was used to prime APTES for coating onto the naturally formed aluminum oxide layer of the AA2024-T3 substrate to minimize the accessibility to humidity during deposition. The APTES-primed AA2024 samples, with an optional additional heat treatment, were then top-coated with PS to form a stacked coating system, which is characterized using EIS in a 3.5% NaCl solution. The surface energy of the coating was estimated from measured static contact angles of two working liquids on the coating. The characterization, together with SEM images of the coating, was analyzed for the causes of failures and explained by four hypothetical mechanisms.

## 2. Materials and Methods

### 2.1. Materials

Bare samples measuring 1 in. × 1 in. were machined from 5 mm thick wrought AA2024-T3 sheets bought from Online Metals. The cut samples underwent sonication in 97% isopropyl alcohol and then in deionized water, each lasting 5 min. Subsequently, the samples were air-dried, baked at 95 °C for 120 min, and left in an oven overnight. The cleaned samples were then stored in a petri dish sealed with parafilm for later use.

Low-molecular-weight, narrowly dispersed PS flakes (Mn 8000, Mw 8800) were purchased from Polymer Source Inc. (product ID 8096-S, Dorval, Canada). Technical-grade acetone of 94% purity was purchased from Sunnyside Corp. (Wheeling, IL, USA). A PS/acetone solution comprising 200 mg of the PS flakes dissolved in 2.28 mL of technical-grade acetone was prepared, resulting in a nominal PS concentration of 10%. APTES with a purity of ≥ 98% was purchased from Millipore Sigma and used without further purification. Milli-Q deionized water and anhydrous ethylene glycol with 99.8% purity (Sigma Aldrich, St. Louis, MO, USA) were used for surface contact angle measurements. For EIS measurements and subsequent in-cell corrosion, a fresh 3.5% NaCl solution was prepared from in-house deionized water and oven-dried, non-iodized, non-fluorinated food-grade NaCl.

### 2.2. APTES Primed on AA2024-T3 Samples

**Figure 1:**
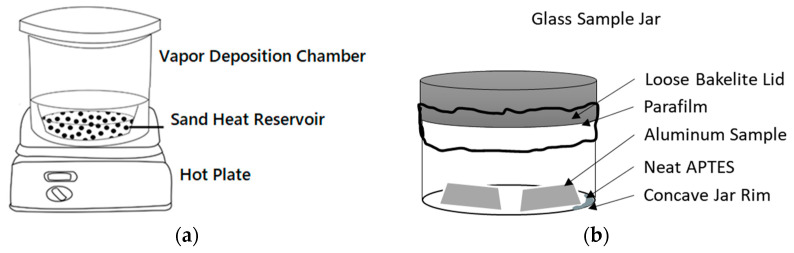
(**a**) Vacuum chamber used as vapor deposition chamber with a metal bowl filled with washed quartz sand; (**b**) 100 mL sample jar for vapor deposition set up for the vacuum chamber.

**Figure 2:**
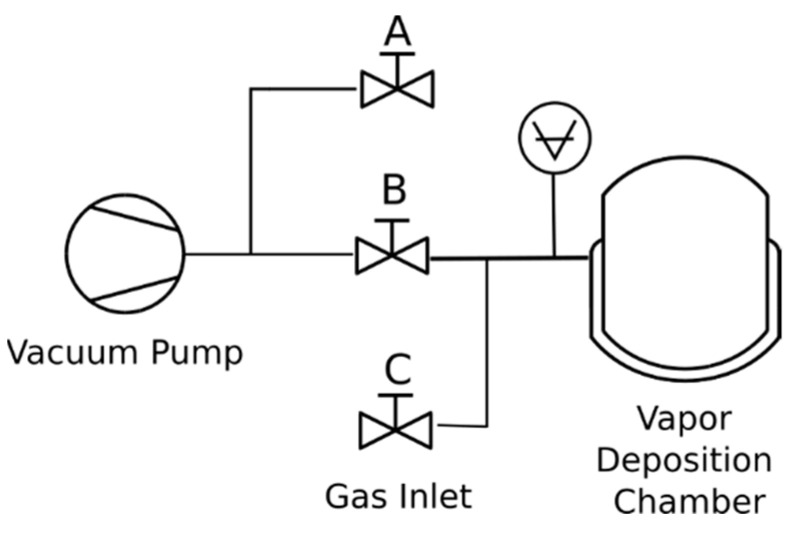
Schematic of vacuum system control.

APTES was applied to the clean AA2024-T3 samples using concentrated vapor deposition. In this process, we employed a heated vapor deposition chamber (Figure 1a) pumped by a dual-stage rotary vacuum pump with the valving system depicted in Figure 2. The chamber undergoes evacuation with valves A and C closed. Once the desired vacuum is attained, valve B is closed to maintain the vacuum in the chamber. To cease pump operation, valve A alleviates backpressure on a rotary vane pump. Additionally, valve C can be optionally used to introduce a dry sparging gas for storage.

The vapor deposition chamber was bedded with industrially crushed quartz sand preheated to 150 °C under a vacuum of 10 mbar. Temperature was monitored with a thermocouple inserted into the vapor deposition chamber, while the chamber itself was heated through a hot plate set at 330 °C under ambient conditions. Afterward, a preheated and dried 100 mL glass sample jar (Figure 1b), holding cleaned aluminum samples and APTES reagent was loosely capped (parafilm was applied to the loose cap to reduce the speed of gas transfer in and out of the container). This jar was then loaded into the heated sand bath in the preheated vapor deposition chamber. While maintaining a temperature of 100 °C throughout the process, the vapor deposition chamber was restored to vacuum conditions under 100 mbar. Under vacuum and heated conditions, the APTES solution was gradually deposited onto the samples from the vapor phase. Three groups of samples placed inside the jar were exposed to the neat APTES within the vacuum chamber for 20, 40, or 60 min, respectively.

Vapor reaction times for this method of coating silanes have been characterized for durations as short as 10 min elsewhere [19]. After completion, the aluminum samples were covered and left undisturbed overnight under ambient conditions. One-third of the samples was reserved for contact angle analysis. The remaining samples were used for the subsequent top-coating with the PS/acetone solution.

### 2.3. Polystyrene Top-Coated on APTES

Half of the remaining APTES-primed samples were spin-coated with 100 µL of acetone containing a 10% PS/acetone solution. The other half of the samples were prepared with further heat treatment and allowed to incubate at an elevated temperature under a pressure of 200 mbar for an additional 24 h to cure the APTES network further before being spin-coated with the 10% PS/acetone solution.

An Ossila brand E441 Spin Coater was programmed to apply 1500 rpm for 5 s, followed by 2000 rpm for another 5 s, and ending with 3000 rpm for 15 s. The coating solution was applied before the conclusion of the first step of the spin-coating program. After the spin-coating process, all the samples underwent 24 h of drying.

### 2.4. Sample Labeling

The samples in this experiment were given a three-digit code: “1-Y-Z”. For samples primed with APTES vapor deposition for 20, 40, or 60 min, the “Y” values are 20, 40, and 60, respectively. Samples are further categorized based on the presence of polystyrene according to the label “Z”, where 0 denotes those only primed with APTES, 1 denotes those with polystyrene top-coated on the “as-is” primed APTES, and 2 denotes those with polystyrene top-coated on the primed APTES that underwent further heat treatment.

### 2.5. Surface Characterization

The sample surfaces were characterized through contact angle measurements with a Rame-Hart goniometer using ethylene glycol and Milli-Q water. The sample lay flat on the surface of the goniometer stage, and 10 µL droplets were manually dispensed out of a 100 µL microsyringe onto the sample surface. The initial contact angle was recorded using DROPimage software (Standard Edition) (Ramé-Hart Instrument Co., Succasunna, NJ, USA). This measurement was replicated across the sample to determine a statistically representative sample.

Exploratory Scanning Electron Microscope (SEM) images were taken using an FEI Quanta200 system in low-vacuum mode. Image processing and scaling were carried out using the freely distributed GNU Image Manipulation Program (GIMP) software (gimp.org, version 2.10.36). All scaling was completed using the pixel length of the SEM Scaling bar.

### 2.6. Electrochemical Impedance Measurement

EIS was employed using a Gamry G300 potentiostat and the Gamry Echem Analyst 6.33 software (Gamry Instruments Inc., Warminster, PA, USA). Measurements were taken using a conventional three-electrode setup in a horizontal flat cell. A graphite counter electrode and Ag/AgCl/saturated KCl reference electrode were used. The sample was mounted in a 1 cm diameter, 0.78 cm^2^ area window. The EIS data were imported into the Python module “impedance.py.” The details of the data-fitting procedure are beyond the scope of this paper and will be reported separately, but a summary is given in the Supplementary Materials.

**Figure 3:**
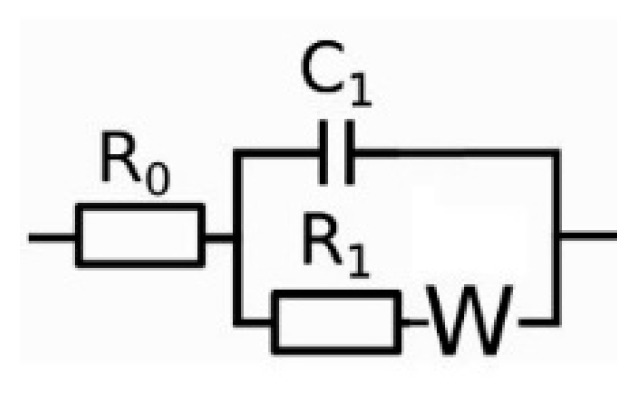
Equivalent circuits for analyzing EIS data: Randles model.

The EIS data of the coated samples were analyzed using a Randles circuit (Figure 3), which contains one time constant featuring two parallel passive elements, C_1_ and R_1_. Here, C_1_ is a constant phase element (CPE) of the form $\frac{1}{Qs^{n}}$ used to account for non-Faradaic charge transfer, where Q and n are the CPE parameters; the resistance R_1_ represents the impedance of the Faradaic current flows. A Warburg element was connected to R_1_ to account for any diffusion-limiting reactions.

## 3. Results and Discussion

### 3.1. APTES Primer

**Figure 4:**
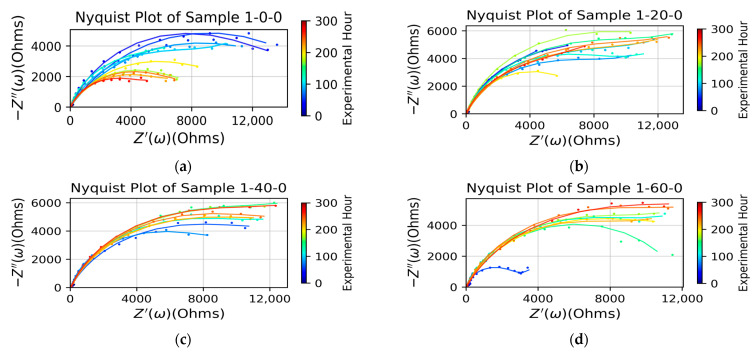
Nyquist plot of measured impedance data (dot) and EIS-fitted trendline over the duration of testing. (**a**) Bare sample and samples coated with a thin layer of APTES vapor-deposited for (**b**) 20 min, (**c**) 40 min, and (**d**) 60 min. All figures share an identical colormap legend regarding timing (in hours), starting from the beginning of an experimental measurement.

Figure 4 compares the impedance between the bare sample and three types of samples primed with APTES vapor deposition for 20, 40, and 60 min, respectively. In the initial corrosion stage, roughly within the first 100 h, the impedance of the bare sample exceeded that of all APTES-primed samples. This behavior can be attributed to the presence of aluminum oxide and its interaction with the vapor-deposited APTES primer when exposed to the aqueous electrolyte solution, as elaborated in the Discussion section. Among the three tested APTES-primed layers, samples -20- and -40- exhibited similar and superior performance compared to the -60- sample.

In the later stage (after the 100 hr mark), the impedance of the bare sample decreased quickly and persistently, indicating that its alumina protection layer was damaged by corrosion. However, all the APTES-primed samples, regardless of their type, enabled larger impedance than the bare sample in this stage. The impedance of the APTES coating per -60- persistently increased over time; in contrast, the -20- and -40- coating layers began to lose their impedance in the middle of the corrosion period tested. The data points distributed in the Nyquist plots of all the APTES-primed samples exhibit one time constant, evidenced by the semicircle arc fitted to each set of the points. Some curves ended up with a feature of Warburg impedance in the lower-frequency regime, signaling the onset of a reaction under partial or complete mass transport control via diffusion [20].

**Figure 5:**
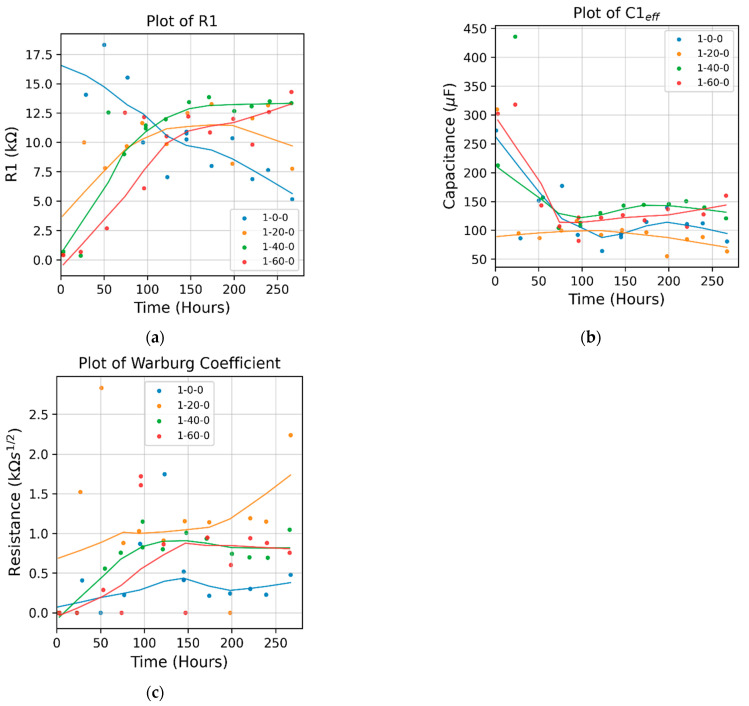
The Randles circuit model’s fitted parameters (dots) and their trendline for APTES-primed samples. (**a**) Coating resistance R_1_. (**b**) Effective capacitance of CPE. (**c**) Warburg impedance. Trendlines were determined using locally weighted scatterplot smoothing (LOWESS) via the statsmodel.py package.

**Figure 6:**
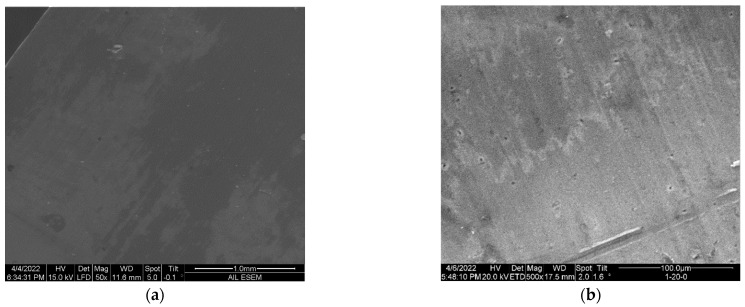

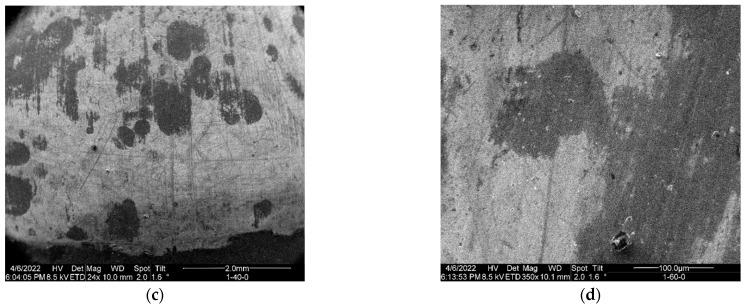
SEM imaging of (**a**) bare sample and APTES-primed samples: (**b**) 1-20-0. (**c**) 1-40-0. (**d**) 1-60-0.

Figure 5 compares the fitted parameters of the Randles circuit model. The model shows that the resistance of the bare sample decreased monotonically over time, indicating the dissolution of the naturally formed oxide layer in the corrosion solution. For the APTES-coated samples, the following was revealed:

- In the first 100 h of corrosion, the 1-20-0 APTES-coated sample exhibited the largest resistance, while the 1-60-0 APTES coating had the least. The effective capacitance (as calculated via $Q\omega^{n}$) of the 1-20-0 sample remained relatively constant from the beginning of corrosion; in contrast, that of the 1-40-0 and 1-60-0 samples decreased over time. (Refer to the individual plots of Q and n in Figure S1 in the Supplementary Materials.) Together with the trendlines in the resistance and capacitance curves, it can be deduced that the change in the modes of electrons and or ion exchange involved in any chemical reactions involving the corrosion and dissolution of the 1-20-0 APTES layer is dictated by the change in resistance, i.e., the Faradaic process.
- After four days of exposure to the corrosion solution, the resistance in the 1-40-0 and 1-60-0 samples increased slightly, which may have been because the coating remained intact while the accumulation of the corrosion products narrowed its porous channels. The resistance of the 1-20-0 sample slightly decreased, implying that the APTES coating in the 1-20-0 sample started to degrade.
- Over the entire corrosion period, the 1-20-0 sample also exhibited a significantly greater Warburg impedance than the other samples shown in Figure 5c. This suggests that the APTES coating prepared as per the -20- protocol enabled a local, interfacial environment that could effectively impede the diffusion of reactive species for charge transfer.
- Figure 6 shows the SEM images of a cleaned bare sample and the samples primed with APTES in the three conditions outlined herein. Note that the SEM imaging of the samples was completed in an exploratory manner to highlight that (1) the commercially wrought surface was not flat but instead exhibited a micro texture caused by the milling process, whereas analytically flat samples (silica or silica oxide) were used for the compared surfaces, and (2) the APTES-primed layer was too thin to cover the manufacturing-induced surface defects of the aluminum samples (e.g., Figure 6d).

### 3.2. Polystyrene Top-Coated on APTES

**Figure 7:**
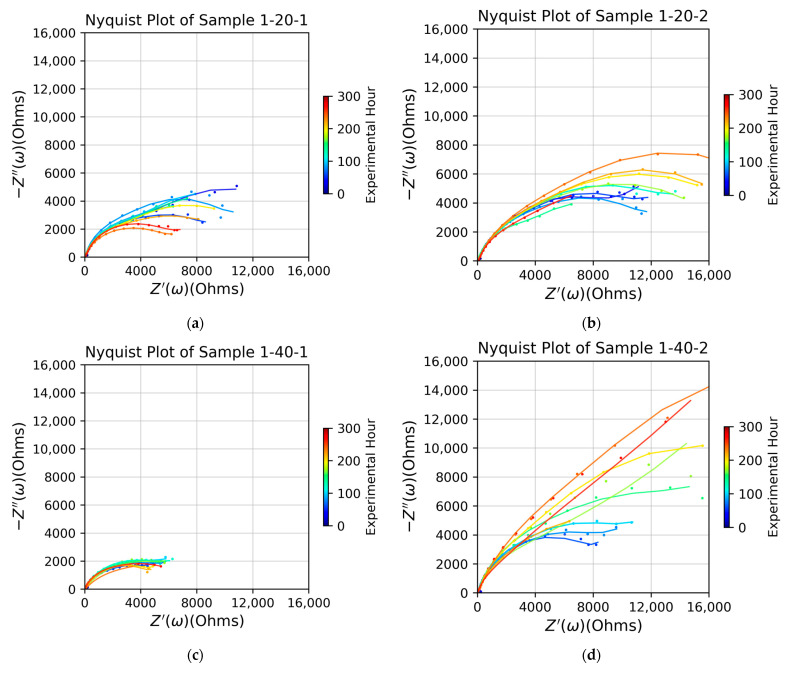

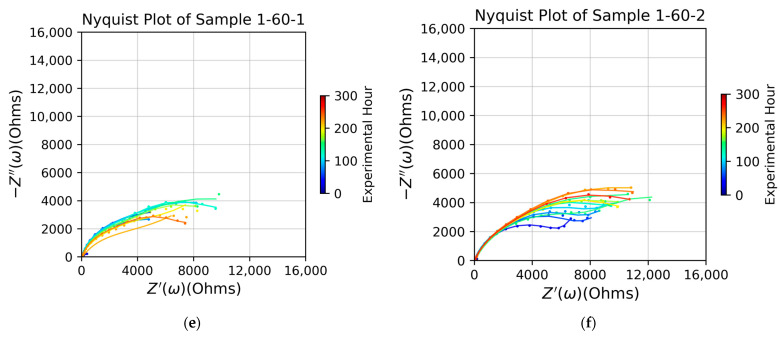
Impedance of PS top-coated samples with the APTES primer cured without further treatment (**a**,**c**,**e**) and with a further heat treatment (**b**,**d**,**f**). Each APTES primer was vapor-deposited for 20, 40, or 60 min, as indicated by the labels of -20-, -40-, or -60-, respectively.

The influence of the primed APTES layer on the impedance of the PS/APTES-coated samples can be seen by comparing the evolving behaviors of the impedance curves of the coated samples in Figure 7.

The samples labeled as 1-Y-1 (Y is 20, 40, or 60) (Figure 7a,c,e) were primed with APTES that had only been cured under the ambient conditions without additional heat treatment. Although the APTES layer primed via vapor deposition for 20 min or 60 min had more significant impedance than the 1-40-1 sample, it degraded over time and thus became hydrolytically unstable, an effect related to partially networked silanols [21].

In the other group of the samples (Figure 7b,d,f), labeled as 1-Y-2 (Y is 20, 40, or 60), the APTES layer had been further heat-treated before the PS layer was top-coated. This group of samples exhibited higher impedance and better hydrolytic stability than the first group (Figure 7a,c,e), which did not undergo additional heat treatment after their APTES layer was polymerized. These results suggest that the post-silanization heat treatment of the APTES layer, following its curing, enhanced networking tightness and hydrolytic stability. This improvement likely occurred due to the removal of any residual water from silanized structures [22]. Heat treatments (post-annealing) may also reduce the adsorbed solvent [4], facilitating condensation and resulting in denser silanol bonds within the networked structure. This group samples all showed increasing impedance over time. For each sample in this group, the diffusion-limiting transport of reactive specimens was evidenced by the upright tail of the impedance curves associated with the 100 h (or earlier) legend. This indicates that over the first 100 h, the electrolyte solution permeated the coating layer, reaching the coating/substrate interface, giving rise to a local environment and thus allowing the reactive species to react with the metal substrate; such species-exchange passages will become narrowed once the corrosion products are stuck at the reaction sites because their removal (likely via diffusion) is impeded by the insoluble coating, despite the porous structure of the coating. Once the corrosion products had accumulated locally at the coating/substrate interface, the sample became less conductive, as shown by the curves of increasing impedance after the 100 h mark. The APTES/PS coating in sample 1-40-2 performed significantly better in these samples because of its relatively higher impedance.

The samples that were primed with APTES via vapor deposition for 40 min (the 1-40-Z samples, where Z is 1 or 2) held their impedance persistently with a minimum extent of degradation, as shown by the monotonically increasing trend of the impedance curves. This implies that the 40 min primed APTES layer cured with the densest network, preventing the loss of its polymerized structure to re-hydrolyzation [23].

### 3.3. Surface Characterization

The dispersion and polar components of the surface energy on the cured APTES surface were calculated by applying the Fowkes model with the measured contact angles of water and ethylene glycol [24,25]. Table 1 lists the properties of water and ethylene glycol used for the calculations. The properties of acetone are also listed to allow for the determination of the wettability of the PS/acetone solution on the sample surface.

Table 2 lists the measured static contact angles and the calculated surface energy. Typical images of the measured contact angles are listed in the Supplementary Materials, Figure S2. Despite a preference for using dynamic contact angle measurements for a comprehensive wettability analysis, static contact angle measurements were taken due to equipment limitations. The statistics were developed through multiple tests conducted on fresh areas of the same sample. All statistics were derived from a sample space of more than 10 measurements. The wide statistical variations presented are due to the non-homogeneous, non-uniform nature of the wrought aluminum sample surface. It is crucial to acknowledge that achieving analytical precision in contact angle measurements for coating wettability necessitates perfectly flat surfaces. However, wrought aluminum sheets do not have these characteristics.

The surface energy of the APTES-primed surface that underwent an additional heat treatment was interpolated and listed in Table 2. Assuming surface energy conservation to be a linear combination of the weighted energies of constituent parts and considering PS characteristics as being independent of the coated surface, the heat-treated APTES without PS can be described as having an energy similar to the 1-20-Z samples plus the difference between the 1-Y-1 and 1-Y-2 samples.

The results in Table 2 reveal the following:

- The measured contact angles of DI water on the cured APTES surface and dried PS surfaces are statistically indistinguishable. However, the contact angle of ethylene glycol on the cured APTES surface decreased with the increase in the vapor deposition time of coating APTES.
- On the APTES-primed surface, the dispersion energy increased over an increasing duration of vapor deposition of the APTES layer. All the APTES-primed samples except for 1-20-0 were characterized by surface energy with a larger dispersion component than the polar component.
- On the top-coated PS surface, the total surface energy calculated for the 1-Y-2 samples (in which the surface of the cured APTES layer had undergone additional heat treatment before being top-coated with PS) was 50% or higher than that of the 1-Y-1 samples (in which the cured APTES layer did not undergo additional heat treatment). Both groups of samples have much smaller dispersion energy. The polar component of the surface energy was relatively smaller than the dispersion component, except for the 1-20-1 sample surface. Note that among the PS-coated samples, the 1-40-2 samples had the greatest surface energy.

**Figure 8:**
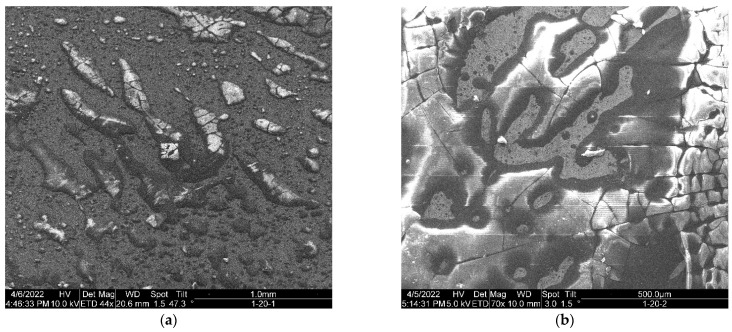

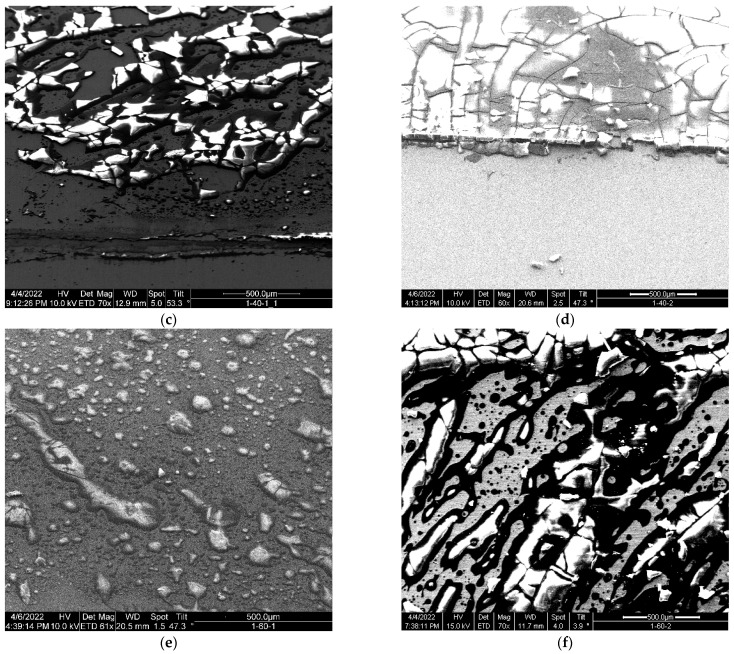
SEM images of dewetted PS patterns on APTES for the samples: (**a**) 1-20-1, (**b**) 1-20-2, (**c**) 1-40-1, (**d**) 1-40-2, (**e**) 1-60-1, and (**f**) 1-60-2.

The SEM images of the APTES-primed samples top-coated with PS in Figure 8 show the heterogeneity of the coating, with various dewetted patterns formed on every PS-top-coated sample. Consistent patterns in PS dewetting were difficult to discern because of the high coating heterogeneity. Cracks in the coating surface can be observed in all the types of PS-coated samples. The crazing is consistent with low-weight PS, which does not have the tensile strength of polymers above the molecular entanglement weight. Small PS dewetted patterns such as “microdots” of approximately 2–3 µm can be observed on all the samples (also shown in the Supplementary Materials, Figure S3). The dewetted patterns in the 1-Y-2 samples constitute a less dense distribution of small patterns and larger packs with crazing.

The top-coated PS layer covered the underneath of the APTES-primed layer (see Supplementary Materials, Figure S4). However, the top coating was not uniform, as the dried PS had dewetted into various shapes, wherein crazing is apparent in some large patterns. The 1-40-2 sample had a relatively uniform top coat, as shown in Figure 8d.

## 4. Discussion

In the context of the various failure scenarios, the naturally formed aluminum oxide, silane primer, and PS top-coating interact with one another in the coating system. The interactions may minimize ion exchange during corrosion and generate undesired local environments, potentially compromising the individual effectiveness of each material as a corrosion barrier. A few mechanisms derived from the experimental results are discussed below with regard to their interactions in the related failure modes. Note that these mechanisms may need further adjustments before they are applied to explain the roles of APTES when not prepared using the concentrated vapor deposition method used herein [4,28].

### 4.1. The Role of APTES in Interaction with the Aluminum Oxide Substrate

APTES was applied to AA2024-T3 through concentrated vapor-phase deposition in a heated, high-vacuum environment with negligible humidity. The hydrolyzation and condensation of ethoxy groups are impeded in the absence of water, resulting in a networked APTES primer that will primarily rely on hydrogen bonds. As a result, the cured APTES primer in the samples was expected to have a structure with a lower density of covalent silanol bonds. However, a more desirable APTES network would feature a higher density of these bonds to grant it better hydrolytic stability as a barrier layer. It can thus be gleaned that during the initial stage (the first 100 h of soaking in the corrosion solution), the naturally formed aluminum oxide layer served as the primary barrier for the corrosion protection of AA2024-T3, because the primed APTES layer is less hydrolytically stable, keeping in mind the three mechanisms related to the reaction of APTES and its interaction with the aluminum oxide substrate developed.

- Mechanism 1: APTES under the effect of water adsorption and permeation

Water access becomes possible when primed samples are later exposed to ambient humidity or immersed in aqueous electrolytes. This leads to the absorption of water molecules on the APTES surface, gradually permeating into the networked APTES layer. This process will then promote hydrolysis and condensation reactions. These water-induced reactions form the desired silanol bonds that can contribute to yielding an improved structural integrity of the APTES layer. However, the permeation of water through the APTES network is a slow process.

- Mechanism 2: APTES reacts with aluminum oxide

Secondly, APTES also reacts with aluminum oxide exclusively by forming stable siloxy bonds (M-O-Si) [29]. This reaction helps reduce the density of unbonded voids or hydrogen bonds susceptible to water attack.

- Mechanism 3: Loss of the naturally formed aluminum oxide to APTES hydrolyzation

However, the APTES-primed layer also facilitates the dissolution of the naturally formed aluminum oxide. The hydrolyzation of the amino groups in APTES makes the solution locally alkaline [30], and aluminum oxide’s solubility changes in alkaline solutions [31].

These three mechanisms competed with one another to collectively determine the overall anti-corrosion performance of the APTES-primed AA2024-T3 samples, which were under the stacked barrier of the natural Al_2_O_3_ layer and the APTES-primed layer. The reason why the 1-20-0 APTES-coated sample exhibited the highest resistance (Figure 4b–d) can be attributed to the following factors:

- The reduced extent of alkaline conditions resulting from hydrolyzation in the APTES layer of the 1-20-0 sample contributed to the increased stability of the aluminum oxide layer.
- The hydrostatic stability of APTES [4]—the APTES primer on the 1-20-0 sample—demonstrated relatively robust hydrolytic stability, as indicated by the consistent evolving behavior of the effective capacitance in Figure 5b. Consequently, the structural integrity of the APTES primer on the 1-20-0 sample remained the most pronounced, resulting in the minimal release of OH- ions and the local solution becoming less alkaline.

This behavior can also clarify why the 1-60-0 APTES coating had the smallest impedance during the initial 100 h of corrosion. The hydrolyzation in the 1-60-0 APTES layer fostered a more potent alkaline environment, leading to increased dissolution of aluminum oxide. Meanwhile, as an APTES layer produced by concentrated vapor deposition is typically only tens of angstroms thick [6] and becomes even thinner after hydrolysis [17], the role of thickness in the APTES layers of the three samples (-20-, -40-, and -60-) in providing barrier protection becomes less significant compared to that of the naturally formed aluminum oxide layer.

### 4.2. The Role of APTES in Interaction with the Top Coating

The extent and uniformity of PS coverage play a crucial role in determining impedance. A PS/acetone solution was applied as a topcoat over the primer, a cured APTES layer in this study. The following mechanism is suggested to explain the effect of the surface energy of a primed APTES in the extent of PS-coated coverage and its relation to the impedance of a sample with the naturally formed aluminum oxide, APTES primer, and PS topcoat.

- Mechanism 4: The surface energy of primed APTES vs. the surface tension of the PS solution.

The PS/acetone solution partially wets the APTES surface; once the PS layer was dried after the acetone had evaporated, various dewetted patterns of dried PS formed, as shown in Figure 8 (and Figures S3 and S4 in the Supplementary Materials). The extent of the wettability of the APTES-primed surface via the PS/acetone solution was determined by the competition between the surface tension of acetone and the surface energy of the cured APTES. Because the PS/acetone solution has a lower surface tension than the surface energy of the cured APTES (refer to Table 1 and Table 2), the PS/acetone solution can wet the APTES-primed surface regardless of the duration of the vapor deposition. The higher the surface energy of the APTES primer surface, the more wetted it will be by the PS/acetone solution. Both observed trends can be related to the fact that a substrate with higher surface energy tends to be more wetted by a liquid with lower surface tension. Enhanced wetting results in fewer dewetting artifacts or patterns. This relationship was illustrated by Ashley et al. [32], who demonstrated that the number density of dewetted polystyrene patterns is proportionate to the surface energy of the cured APTES on which the PS was deposited. The textural roughness and heterogeneity of the wrought sample surface also facilitate wettability. Because the surface tension of the PS/acetone solution is less than the surface energy of the primed APES surface, a higher surface energy of the primed APTES promotes a larger extent of wetting via the PS/acetone solution.

From Mechanism 4, it can be surmised that an APTES primer with a higher surface energy (which can be induced with an additional heat treatment) can be wetted to a greater extent by the PS/acetone solution. The dried PS layer from such an APTES surface with higher energy will form larger patterns [32]; however, given that the chosen PS with low molecular weight has less tension, the dried PS layer can easily be cracked. This can explain why the 1-Y-2 samples exhibited dewetted patterns consisting of larger packs with crazing.

### 4.3. Potential Applications for Micro Devices

The vapor-phase deposition of silanes is applicable in the photo-lithography-based microfabrication process [33]. As described in this paper, this technique holds significant potential for a wide range of applications in microfabricated devices requiring the bonding or adhesion of dissimilar materials [34]. These applications include hermetic sealing, chemical sensing, and the modification of the mechanical characteristics of microstructures. Several practical applications are enumerated below.

Firstly, a conformal polymeric topcoat on a microdevice can effectively address inherent stress-induced warpage. This intrinsic stress arises from thermal effects caused by the selective removal (etching) of dissimilar materials from each thin film individually deposited at different temperatures on a silicon wafer during fabrication. This thermal stress distorts patterned features, altering their intended dimensions, and can cause warping in micro cantilevered structures [35]. To mitigate this warpage, Kuchiji et al. addressed the issue using a polyurethane coating [36]. Polyurethane, more commonly used than polystyrene, exhibits a relatively strong elasticity modulus, enabling a thin coating to counteract intrinsic thermal stress and reduce warpage in microdevices. The durability of the polyurethane coating on vibratory microstructures is crucial and can be enhanced via the APTES-modification of polyurethane [37]. This modification involves directly mixing a low dose (e.g., 1%) of concentrated APTES with waterborne polyurethane or using APTES as a primer for top coating with 2K polyurethane solutions.

Secondly, an APTES-modified surface exhibits hydrophobic behavior (as indicated in Table 2), making a microsensor chip water/moisture repellent [38].

Thirdly, for microsensors requiring a biocompatible or hermetic seal for chronic implantation, a conformal coating of low-Young’s-modulus polymers can preserve the designed sensing capability [36,39]. To ensure strong adhesion and durability of the added coating for a long-term deployment, silanes like APTES can serve as a primer for top-coating with Parylene for in vivo applications [40] or be mixed with polyurethane as a hybrid composite for ex vivo applications [41]. In contrast to using strong polyurethane to modify the mechanical behavior of a microdevice, polymers with a low elasticity modulus like Parylene C can be coated on an APTES-primed surface for encapsulation so that further patterning will not alter the intended mechanical impedance of microdevices [36,39,42]. Another low-elastic-strength polymer, polyimide (Kapton), is commonly used in poly(dimethylsiloxane)-based soft lithography with APTES as the bonding agent [43,44]. These applications are all rooted in the use of silanes, with a specific focus on APTES as an adherent bonding agent or a surface modifier for top coating with a polymer of choice to serve various purposes.

## 5. Conclusions

The complexity of the arrangement of and interactions among the three layers of the naturally formed aluminum oxide, primed silane, and top-coated PS film make the stacked coating system difficult to manage for predictable anti-corrosion performance.

In this paper, we addressed this challenge by examining the role of the silane primer APTES, commonly used as an adherent agent for inorganic/organic bonding. For a primed layer made via concentrated vapor deposition, the findings indicate that an APTES primer of an optimal thickness (as in the 1-40-2 sample) offers the best corrosion resistance. This result is attributed to the relatively large surface energy in this primed APTES surface that enables better wettability via the PS solution and, consequently, more uniform coverage of the dried PS topcoat. During the early exposure to the corrosion solution, a thinner APTES primer would enhance protection against corrosion. This can be explained by the mechanisms of hydrolytic stability and the interaction between the hydrolyzation/condensation in soaked APTES and the dissolution of the naturally formed aluminum oxide pre-existing in the bare samples. An APTES primer subjected to an additional heat treatment before top coating always performs better. Finally, the applications of APTES and silanes in micro devices were projected.

## Figures and Tables

**Table 1:** Polar and dispersion parts of the surface energy of the working liquid agents used.

Solvent	Mol. Formula	CAS #	Surface Energy/Tension (mJ/m^2^)			Reference
			γ_l_	Dispersion γ^D^	Polar γ^p^	
DI Water	H_2_O	7732-18-5	72.8	26.85	45.9	[26]
Ethylene glycol	C_2_H_6_O_2_	107-21-1	48.0	29.0	19.0	[26]
Acetone	(CH_3_)_2_CO	62-53-3	24.5	--	--	[27]

**Table 2:** Measured contact angles and calculated surface energy components.

Sample (1-Y-Z)	Contact Angle (°)		APTES		PS	Surface Energy (mJ/m^2^)		
	DI Water	Ethylene Glycol	Vapor Deposition Time (min)	Additional Heat Treatment	Top-Coated	Dispersion γ_s_^D^	Polar γ_s_^p^	Total γ_s_^p^ + γ_s_^D^
1-0-0	82 ± 21	65 ± 12	--	--	--	13.3	11.1	24.4
1-20-0	63 ± 11	47 ± 14	20	NO	NO	9.6	29.6	39.2
1-40-0	63 ± 10	34 ± 8	40	NO	NO	23.1	17.0	40.2
1-60-0	60 ± 12	22 ± 16	60	NO	NO	29.6	15.2	44.7
1-20-0 *	47	56	20	YES	NO	43.3	15.9	59.2
1-40-0 *	48	57	40	YES	NO	46.6	13.9	60.5
1-60-0 *	53	62	60	YES	NO	50.7	10.1	60.8
1-20-1	81 ± 14	66 ± 12	20	NO	YES	10.7	13.8	24.4
1-40-1	83 ± 6	56 ± 5	40	NO	YES	29.6	3.5	33.1
1-60-1	83 ± 6	59 ± 15	60	NO	YES	24.3	5.1	29.4
1-20-2	90 ± 7	57 ± 5	20	YES	YES	44.4	0.1	44.5
1-40-2	94 ± 8	58 ± 7	40	YES	YES	53.1	0.3	53.4
1-60-2	93 ± 8	60 ± 1	60	YES	YES	45.4	0.0	45.4

* Interpolated data.

## Data Availability

Raw EIS data can be found at https://sites.google.com/a/alaska.edu/cf-chen/home/organic-inorganic-coating.

## Supplementary Materials

The following supporting information can be downloaded at https://www.mdpi.com/article/10.3390/mi15010093/s1. 1. Summary of data fitting using the Python Module “impedance.py”. Figure S1: Fitted CPE parameters. Figure S2: Images of water contact angles measured on the individual substrate surfaces. Figure S3: SEM images of top-coated PS, dried, on the APTES-primed samples. Figure S4: SEM images of dewetted PS patterns on APTES with an articulately made edge or groove.
