# Supplementary material for: The Role of APTES as a Primer for Polystyrene Coated AA2024-T3
Source: Micromachines (Basel). 2023 Dec 31;15(1):93. doi: 10.3390/mi15010093 (PMC10820139; doi:10.3390/mi15010093)
Supplement: Supplementary file 1 [file micromachines-15-00093-s001.zip › micromachines-2792378-supplementary.pdf]

micromachines

Supplementary Materials

The Role of APTES as a Primer for Polystyrene Coated AA2024-T3

John Halford IV and Cheng-fu Chen *

Department of Mechanical Engineering, University of Alaska Fairbanks, Fairbanks, AK 99775-5905, USA; jhhalfordiv@alaska.edu
* Correspondence: cchen4@alaska.edu; Tel.: +1-(907)-474-7265

Abstract: (3-Aminopropyl)triethoxysilane (APTES) silane possesses one terminal amine group and three ethoxy groups extending from each silicon atom, acting as a crucial interface between organic and inorganic materials. In this study, after APTES was deposited on the aluminum alloy AA2024-T3 as a primer for an optional top coating with polystyrene (PS), its role with regard to stability as a protection layer and interaction with the topcoat were studied via combinatorial experimentation. The aluminum alloy samples primed with APTES under various durations of concentrated vapor deposition (20, 40, or 60 min) with an optional post heat treatment and/or PS topcoat were comparatively characterized via electrochemical impedance spectroscopy (EIS) and surface energy. The samples top-coated with PS on an APTES layer primed for 40 min with a post heat treatment revealed excellent performance regarding corrosion impedance. A primed APTES surface with higher surface energy accounted for this higher corrosion impedance. Based on the SEM images and the surface energy calculated from the measured contact angles on the APTES-primed surfaces, four mechanisms are suggested to explain that the good protection performance of the APTES/PS coating system can be attributed to the enhanced wettability of PS on the cured APTES primer with higher surface energy. The results also suggest that, in the early stages of exposure to the corrosion solution, a thinner APTES primer (deposited for 20 min) enhances protection against corrosion, which can be attributed to the hydrolytic stability and hydrolyzation/condensation of the soaked APTES and the dissolution of the naturally formed aluminum oxide pre-existing in the bare samples. An APTES primer subjected to additional heat treatment will increase the impedance of the coating system significantly. APTES, and silanes, in general, used as adherent agents or surface modifiers, have a wide range of potential applications in micro devices, as projected in the Discussion section.

Keywords: vapor deposition; coating; primer; polystyrene; APTES; silanes; AA2024-T3; corrosion; electrochemical impedance spectroscopy; surface energy

Citation: Halford IV, J.; Chen, C.-f. The Role of APTES as a Primer for Polystyrene Coated AA2024-T3. *Micromachines* **2024**, *15*, 93. https://doi.org/10.3390/mi15010093

Academic Editor: Amir Hussain Idrisi

Received: 13 December 2023
Revised: 26 December 2023
Accepted: 29 December 2023
Published: 31 December 2023

Copyright: © 2023 by the authors. Submitted for possible open access publication under the terms and conditions of the Creative Commons Attribution (CC BY) license (https://creativecommons.org/licenses/by/4.0/).

1. Summary of Data Fitting with Python Module "impedance.py"

The low-frequency portions of the spectra, demonstrating a deviation of more than 5% from a linear KK-transform, were systematically truncated. Subsequently, models representing oxide, coating, and Randles cells were fitted, with their capacitors combinatorically replaced by CPE elements. Additionally, a single Warburg element was introduced in series with one resistor for each model.

The impedance circuits that demonstrated the best fit across all samples and analyses for various time periods were carefully selected to advance the analysis. To mitigate the risk of overfitting and evident misfitting, constraints were applied to the solution resistance. In experiments involving two CPE elements, a deliberate effort was made to create asymmetry and distinctiveness between them. Analyses featuring a capacitance exceeding 5 mF were systematically discarded.

2. CPE Parameters

The CPE used in the Randles circuit model is of the form $\frac{1}{Qs^n}$. The fitted parameters are shown in Figure S1 in which the magnitude of Q is referred to the label "CEP1".

3. SEM Images of Topcoated PS

Figure S3 compares the dewetted PS patterns.

Figure S4 shows that the dried PS topcoating that covers the underneath APTES layer, as evidenced by its observable thickness through the step along the articulately made step or groove. The topcoating is not uniformly; dewetted patterns of various sizes and shapes are evident, and crazing can be observed from some large patterns.

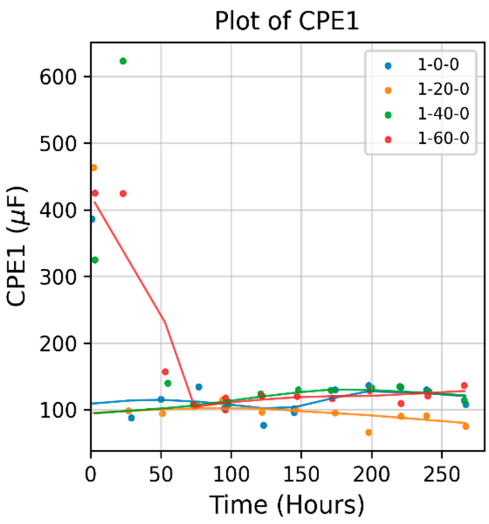

(**a**)

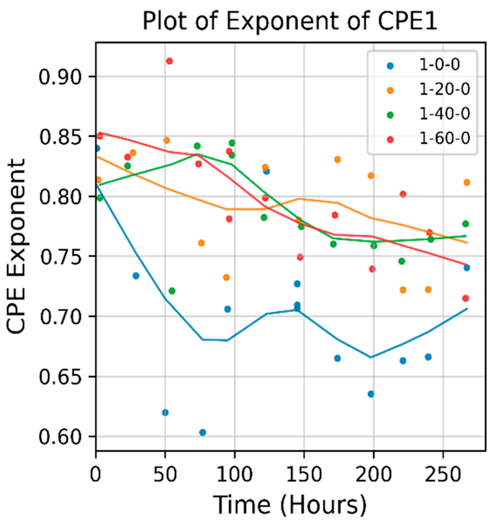

(**b**)

Figure S1. Fitted CPE parameters. (**a**) Capacitance of CPE. (**b**) Exponent of CPE.

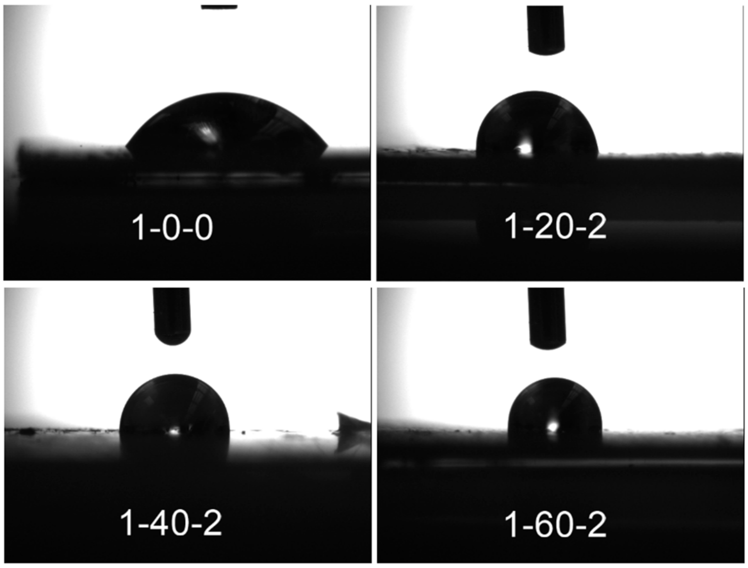

Figure S2. Images of water contact angles measured on the individual substrate surfaces. 1-0-0: bare specimen. 1-Y-2: Topcoat PS on the APTES primed on the sample, whereby the primed layer was deposited for Y = 20, 40, and 60 min individually. (Refer to Section 2.4 for the detail of sample labeling.)

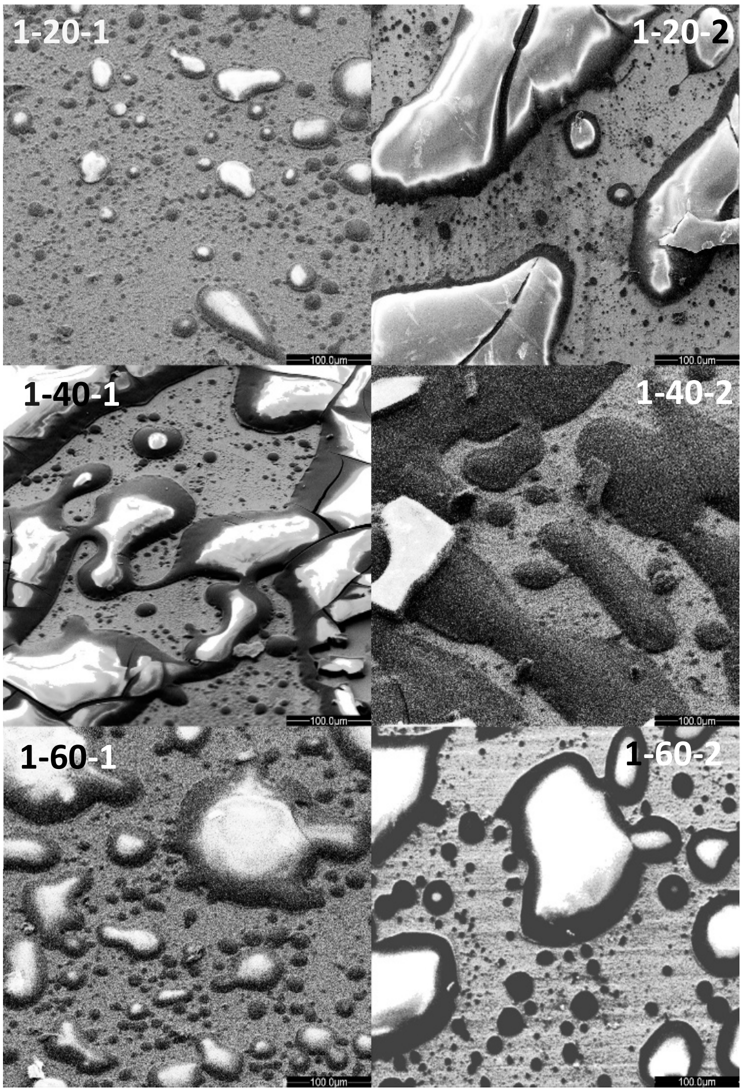

Figure S3. SEM images of top-coated PS, dried, on the APTES-primed samples. The images are resized (and trimmed) to the same scale to compare the dewetted PS patterns.

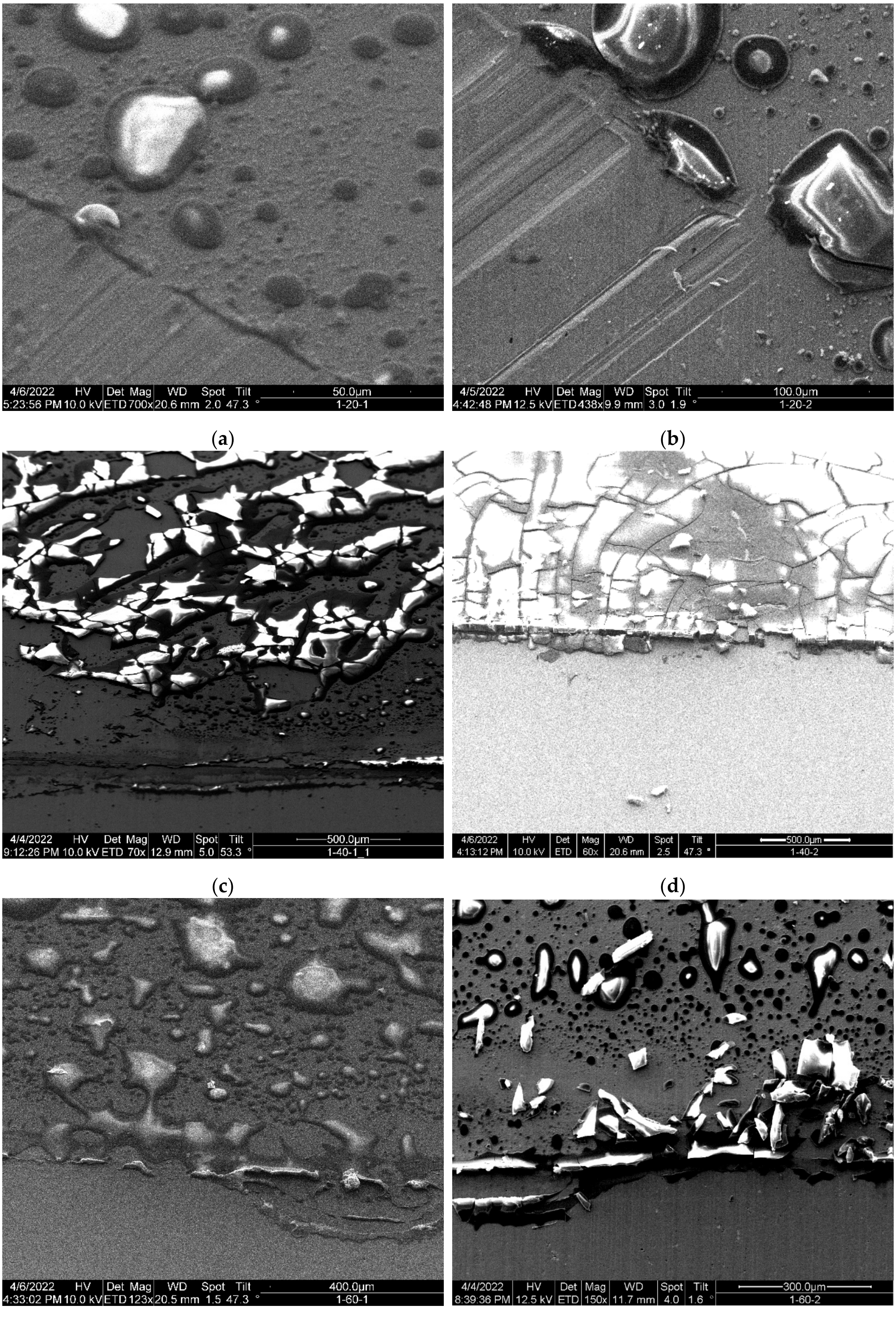

Figure S4. SEM images of dewetted PS patterns on APTES with an articulately made edge or groove. (**a**) 1-20-1, (**b**) 1-20-2, (**c**) 1-40-1, (**d**) 1-40-2, (**e**) 1-60-1, (**f**) 1-60-2.
